# Decreased PPM1B Expression Drives PRMT5-Mediated Histone Modification in Lung Cancer Progression

**DOI:** 10.3390/biom15111581

**Published:** 2025-11-11

**Authors:** Attila Makai, Ilka Keller, Fanni A. Szalmás, Ádám Ungvári, Dániel Horváth, Evelin Major, Attila Enyedi, István Takács, Beáta Lontay

**Affiliations:** 1 Health Care Service Units, Pulmonology Clinic, University of Debrecen Clinical Center, University of Debrecen, H-4032 Debrecen, Hungary; makai.attila@med.unideb.hu; 2 Department of Medical Chemistry, Faculty of Medicine, University of Debrecen, H-4032 Debrecen, Hungary; keller.ilka@med.unideb.hu (I.K.); szalmas.fanni@med.unideb.hu (F.A.S.); hordani89@gmail.com (D.H.);; 3 Health Care Service Units, Department of Surgery, University of Debrecen Clinical Center, University of Debrecen, H-4032 Debrecen, Hungarytakacs.istvan@med.unideb.hu (I.T.)

**Keywords:** planocellular carcinoma, adenocarcinoma, lung cancer, protein arginine methyltransferase 5, protein phosphatases, methylation

## Abstract

Pulmonary carcinoma remains a highly aggressive malignancy driven by complex signaling and epigenetic dysregulation. This study investigates a novel oncogenic pathway involving the Mg^2+/^Mn^2+^-dependent protein phosphatase 1B PPM1B/myosin phosphatase (MP)/protein arginine methyltransferase 5 (PRMT5) axis, which promotes carcinogenesis by symmetrically dimethylating histone H2A and suppressing tumor suppressor genes. We hypothesized that loss of PPM1B would activate this pathway and drive tumorigenesis. Western blotting, PCR, and immunohistochemistry revealed a significant reduction in PPM1B expression in both squamous cell carcinoma (SCC) and human lung adenocarcinoma (ADC) compared to normal lung tissues, which correlated with worse patient survival. Despite an increase in total MYPT1, the regulatory subunit of MP, its inhibitory phosphorylation at Thr853 was significantly elevated in both tumor types. The inactivation of MP corresponded with a significant increase in the activating phosphorylation of PRMT5 at Thr80, especially in SCC, which was linked to a particularly poor prognosis. Downstream, this resulted in a dramatic elevation in the symmetric dimethylation of histone H2A, leading to decreased expression of retinoblastoma protein. Our findings demonstrate that decreased PPM1B expression drives the oncogenic activation of the MP/PRMT5 axis. This mechanism contributes to the aggressive nature of SCC, establishing PPM1B as a promising prognostic marker in lung cancer.

## 1. Introduction

Lung cancer remains a leading cause of cancer-related mortality worldwide [1,2]. The incidence of lung cancer has been the highest among all cancer types worldwide. In 2018, a large fraction of newly diagnosed malignancies, 12% were lung cancer cases [2], and the distribution of disease incidence exhibits considerable heterogeneity [3,4]. Despite significant therapeutic advances, the prognosis remains poor. In case of non-small cell lung cancer, the five-year survival rate is approximately 23%, with particularly low survival observed in squamous cell carcinoma (SCC) compared to adenocarcinoma (ADC) [5,6]. This poor prognosis is largely due to the fact that only 20–30% of lung cancer cases are diagnosed at an early stage. As a result, effective lung cancer screening remains a major challenge. Currently, low-dose computed tomography screening in high-risk populations is the most effective method available. However, it is far from optimal, as substantial heterogeneity exists among studies of its impact on mortality reduction [7,8]. Lung cancer is generally classified into two major types: small cell lung cancer, accounting for approximately 15% of cases, and non-small cell lung cancer, which comprises about 85%. The most common histological subtypes include ADC, SCC, and large cell carcinoma [2,9,10]. Besides the well-known risk factors for lung cancer (e.g., cigarette smoking, air pollution, etc.), numerous genetic mutations (e.g., p. 53) have been identified in the tumorigenesis [11,12]. Among them are the so-called druggable mutations (e.g., *EGFR*, *ALK*), which play a crucial role in treatment [2,13,14,15,16,17]. Moreover, not only somatic mutations but also epigenetic alterations, such as aberrant DNA methylation, contribute to lung cancer development by affecting the expression of regulatory enzymes involved in the cell cycle. These methylation patterns are increasingly recognized as potential diagnostic, prognostic, and predictive biomarkers [18,19].

In cancer development, an imbalance in post-translational modifications (PTMs) such as phosphorylation and methylation also plays a crucial role [20,21]. Protein arginine methyltransferase 5 (PRMT5) symmetrically dimethylates arginine residues in histones and epigenetically controls the expression of an array of target genes by repressing gene expression [22,23]. In addition, PRMT5 also modifies non-histone proteins, including transcription factors like p53, thus playing a role in cancer cell proliferation and transformation [24]. PRMT5 is a frequently studied therapeutic target, and the effectiveness of PRMT5 inhibitors in lung cancer cell lines or xenografts has shown promising results [25,26]. The role of PRMT5 in tumorigenesis is clear, but the PTMs of PRMT5 can also contribute to malignant transformation. It was proved that PRMT5 has a regulatory site at the Thr80 amino acid residue, which, when phosphorylated by Rho A activated kinase (ROK), increases activity, but dephosphorylation by myosin phosphatase (MP)-consisting of a protein phosphatase 1 catalytic subunit (PP1c) and a MYPT1 regulatory subunit [27]-decreases PRMT5 activity in hepatocellular carcinoma cells [28]. The activity of MP is directly regulated by another phosphatase, the Mg^2+/^Mn^2+^-dependent protein phosphatase 1B (PPM1B), which activates MP holoenzyme by dephosphorylating the MYPT1 subunit at the Thr696 and Thr853 inhibitory phosphorylation sites. Phosphorylation of Thr696 and Thr853 sites induces a structural movement in MYPT1 that allows the inhibitory phosphorylation region of MYPT1 to interact with the substrate binding groove and the catalytic center of protein phosphatase 1 catalytic subunit (PP1c) [29]. PPM1B inhibits tumor cell colonization and metastasis by modulating MP and PRMT5; thus, the indirect tumor suppressor nature of MP was verified [29]. The disruption of the PPM1B/MP/PRMT5 pathway, manifested by pathological modifications of the PTMs, leads to tumorigenesis [29]. Since both MP and PRMT5 showed an alteration in their regulatory phosphorylation by Proteome Profiler tissue array screening in several cancer types [28], we aimed to investigate the expression and modifications of the elements of the PPM1B/MP/PRMT5 pathway in different histological types of lung cancer.

## 2. Materials and Methods

### 2.1. Patients

All chemicals were obtained from MilliporeSigma (St. Louis, MO, USA) unless indicated otherwise. Patients presented with a 2 cm or larger pulmonary shadow on chest computed tomography CT, and who were also deemed operable, were evaluated as suitable candidates for surgical intervention by the institutional multidisciplinary pulmonary oncology team. Surgical procedures were performed at the Department of Thoracic Surgery, Surgical Clinic, University of Debrecen. Patients represented with a histologically verified tumor diagnosis were not a selection criterion. Patient data were collected between 12 November 2019 and 25 July 2023, following the GDPR rules and regulations [30] of the University of Debrecen with the ethical permit (34199-L/2019/EKU).

### 2.2. Biopsies and Tissue Samples

During surgery, a segmentectomy or lobectomy was performed. We collected tissue samples from the tumor and from healthy lung tissue outside the infiltration zone (minimum 1 cm), technically as far as possible. From every lobe/segment, three tissue pieces were collected: (1) fresh frozen for Western blotting, (2) in tissue matrix samples that were immediately placed in liquid nitrogen and then stored at −70 °C, and (3) in RNAlater, which were stored at −20 °C. Classification of patients was performed according to the 2015 WHO criteria [9,31]. Stage classification was based on the 8th edition of the TNM (tumor, node, and metastasis) staging system [31]. The location of the tumor by lung lobe was also analyzed [32].

### 2.3. Protein Extraction

The protein extraction was conducted as described before [33]. Briefly, modified RIPA buffer (50 mM Tris (pH 7.4), 0.2% sodium deoxycholate, 0.2% sodium dodecyl-sulfate (SDS), 1% Triton X-100, and 1 mM EDTA supplemented with 1× protease inhibitor cocktail, 1× phosphatase inhibitor cocktail, 1 mM phenylmethylsulfonyl fluoride, and 1 µM microcystin-LR) was added in a 10:1 volume-to-weight ratio and were homogenized using a TissueRuptor. Protein concentrations were determined with Pierce BCA Protein Assay Kit (Thermo Scientific, Waltham, MA, USA) [34].

### 2.4. Western Blot Analysis

Protein lysates (30 µg per sample) were separated by size by SDS-PAGE using 4–20% precast Criterion gels (Bio-Rad Laboratories, Hercules, CA, USA), followed by electrophoretic transfer onto nitrocellulose membranes and processed according to previously published protocols [35]. Supplementary Table S1 provides detailed information on the antibodies used in the Western blot analyses. Protein bands were visualized using WesternBright ECL chemiluminescent substrate (Advansta Inc., San Jose, CA, USA) and imaged with a ChemiDoc Touch Imaging System (Bio-Rad Laboratories, Hercules, CA, USA).

### 2.5. RNA Isolation from Lung Tissue

Total RNA was isolated from lung tissue using TRIzol reagent (Life Technologies, Carlsbad, CA, USA) following a modified manufacturer’s protocol. Tissue samples were homogenized in 200 μL TRIzol using a TissueRuptor homogenizer, and the RNA was extracted as described before [36]. The RNA concentration was determined using a NanoDrop Fluorospectrometer (Thermo Fisher Scientific, Waltham, MA, USA).

### 2.6. Semi-Quantitative Reverse Transcription Quantitative Polymerase Chain Reaction (RT-qPCR) Analysis

Reverse transcription was performed using 1 µg of total RNA per sample with a cDNA Synthesis Kit (Thermo Fisher Scientific, Waltham, MA, USA) at 37 °C for 120 min. The resulting cDNA was used as a template for quantitative PCR, carried out with 2× Xceed qPCR SYBR Green Master Mix (Institute of Applied Biotechnologies, Prague–Strašnice, Czech Republic). Primer sequences are provided in Supplementary Table S2. PCR amplification was conducted on a LightCycler 480 system (Roche Applied Science, Penzberg, Germany) using the following thermal cycling conditions: initial denaturation at 95 °C for 3 min, followed by 50 cycles of 95 °C for 3 s, 60 °C for 30 s, and 72 °C for 90 s. Cp values were normalized to the geometric mean of the housekeeping genes *GAPDH* and *Cyclophilin A* [36].

### 2.7. Immunohistochemistry

Formalin-fixed, paraffin-embedded tissue sections from SCC, ADC, and their corresponding non-tumorous lung tissue counterparts were subjected to immunohistochemistry as described before [35]. Briefly, xylene was used for deparaffinization, antigen retrieval was performed in a pressurized heat chamber, and slides were then immersed in citrate buffer (11 mM, pH 6.0; prepared from 29.4 g sodium citrate and 2.1 g citric acid in 1000 mL distilled water) after alcohol treatment. Non-specific binding was blocked by 5% bovine serum albumin (BSA) in 1× TBST, and the slides were subjected to primary antibody treatments against MYPT1, PRMT5, and PPM1B; horse radish-conjugated secondary antibody control sections were also included. DAB substrate (3,3′-diaminobenzidine) was applied to visualize antibody binding. Counterstaining was performed with hematoxylin-eosin. The chromogenic signal development was visualized using a Leica microscope equipped with a Leica MC120 HD camera (Leica Microsystems GmbH, Wetzlar, Germany). 

### 2.8. Immunofluorescent Staining

Confocal microscopy was performed as described previously [34]. Briefly, tissue sections derived from the tumor and corresponding control lung tissues were subjected to immunofluorescence staining. Cell nuclei were counterstained with DAPI (4′,6-diamidino-2-phenylindole; blue fluorescence). Primary antibodies against MYPT1, MYPT1^pT853^, PRMT5, and PRMT5^pT80^ were applied at a dilution of 1:100, followed by detection with Alexa Fluor 488 and 546-conjugated secondary antibodies. Imaging was performed using a 40× objective of the Leica SP8 confocal microscope (Leica Microsystems GmbH, Wetzlar, Germany).

### 2.9. Kaplan–Meier Survival Analysis

Overall survival [37] and disease-free survival (DFS) [38] were recorded from the time of patient enrollment until July 2024, with survival time expressed in months. Kaplan–Meier curves were constructed to assess survival outcomes in relation to histological subtype and molecular marker expression [37]. Patients with SCC (red) and ADC (black) were stratified based on relative expression levels of MYPT1, PRMT5, H2A, and PPM1B, as well as the relative phosphorylation status of MYPT1^pT853^ and PRMT5^pT80^, and H2A symmetric dimethylation. For each molecular parameter, patients were categorized into “high” and “low” groups using the median value of the entire cohort as a cutoff. Survival differences between groups were analyzed using the log-rank (Mantel–Cox) test, with *p* < 0.05 considered statistically significant. The log-rank test statistic was interpreted based on the Chi-square distribution.

### 2.10. Statistical Analysis

Densitometric analysis of immunoblots was performed using ImageJ software (Version 1.x) (NIH, Bethesda, MD, USA). Quantitative data were visualized as bar graphs generated with GraphPad Prism 8 (GraphPad Software, San Diego, CA, USA), which was also used for all statistical analyses. Phosphorylated protein levels were normalized to their corresponding total protein levels and expressed as relative values. Total protein expression was normalized to the loading control GAPDH and likewise presented as relative values. Data are shown as bar charts, where one bar stands for mean ± standard deviation (SD), and *n* refers to the number of independent biological replicates. Statistical significance was assessed using unpaired, two-tailed *t*-tests for comparisons between two groups, and one-way or two-way ANOVA followed by Tukey’s multiple comparisons test for analyses involving more than two groups. Outliers were identified and excluded based on Grubbs’s test. A *p*-value < 0.05 was considered statistically significant.

## 3. Results

### 3.1. PPM1B Expression Is Significantly Reduced in Both ADC and SCC Samples

**Figure 1:**
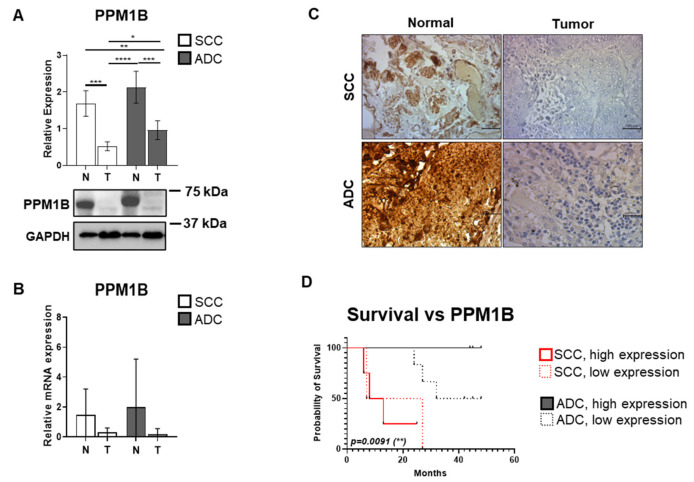
PPM1B expression and its association with survival in squamous cell carcinoma (SCC) and adenocarcinoma (ADC) lung cancer tissue samples. (**A**) Protein lysates from ADC and SCC tumors, along with corresponding non-tumorous control lung tissues, were analyzed by SDS-PAGE followed by Western blot using anti-PPM1B antibody. GAPDH served as a loading control. Relative protein expressions of PPM1B are shown as bar graphs, mean ± SD for *n* = 5–7 per group. The original WB images are shown in Figure S3. (**B**) PPM1B mRNA expression in ADC and SCC control and tumor tissues was assessed by RT-PCR. Cp values were normalized to housekeeping genes GAPDH and Cyclophilin A and presented as relative expression values. Data are depicted as bar graphs (mean ± SD, *n* = 3–4 per group). Statistical comparisons between multiple groups were performed using one-way or two-way ANOVA followed by Tukey’s multiple comparisons test; comparisons between two groups were analyzed using unpaired two-tailed *t*-tests. Statistical significance was defined as *p* < 0.05 (*), *p* < 0.01 (**), *p* < 0.001 (***), and *p* < 0.0001 (****). (**C**) Representative immunohistochemistry images of DAB-stained SCC and ADC tumor and control tissues probed with anti-PPM1B primary antibody. Scale bar: 200 µm. (**D**) Kaplan–Meier survival curves depicting overall survival of patients with SCC or ADC histological subtypes, stratified by PPM1B expression levels. Survival probability is plotted as a function of time (in months) from study enrollment. Patients were categorized by histological subtype (SCC: red; ADC: black), and within each group, samples were further divided into low or high PPM1B expression based on the median value of the entire cohort. Survival curves were compared using the log-rank (Mantel–Cox) test. Statistically significant differences (*p* < 0.01) are marked with an asterisk (**). The log-rank test statistic approximates the Chi-square distribution.

A total of 38 patient samples were collected in this study, and their clinical data were analyzed (Figure S1A–D, Tables S3 and S4). The most common histological types were further analyzed: adenocarcinoma (55.26%) and squamous cell carcinoma (26.31%) (Figure 1A and Table S1). The median overall survival (OS) was 44 months for ADC and 20 months for SCC patients (*p* = 0.0170). The median disease-free survival (DFS) was 17 months in the ADC group and 14 months in the SCC group (*p* = 0.0326). Both OS and DFS were significantly better among patients with ADC. In our previous work, we identified PPM1B as an upstream regulator of the MP/PRMT5/histone signaling pathway, providing a novel mechanism of tumorigenesis in HeLa cells and cervical carcinoma [29]. We analyzed both ADC and SCC tissue samples, using their corresponding normal lung tissues with semi-quantitative Western blot analysis using an anti-PPM1B antibody and GAPDH as a loading control. Figure 1A shows the relative protein expression levels of PPM1B. PPM1B expression showed a reduction in both cancer types by 0.31-fold (*p* = 0.0009) and by 0.45-fold (*p* = 0.0003) in SCC and in ADC, respectively. Moreover, a 0.544-fold (*p* = 0.0285) lower PPM1B expression level was detected in SCC tumor tissue compared to ADC samples (Figure 1A). Our data was further supported by RT-PCR analysis of *PPM1B* mRNA expression (Figure 1B). However, no statistically significant differences in mRNA expression were observed among the groups. Representative immunohistochemistry (IHC) images of DAB-stained SCC, ADC, and their corresponding normal tissues show differential expression of PPM1B. Altered PPM1B expression is evident among the four groups, with a notably stronger DAB signal observed in the normal tissues compared to the carcinoma samples, confirming a higher PPM1B expression in non-cancerous tissue (Figure 1C).

To evaluate the prognostic relevance of PPM1B expression, SCC and ADC patients were classified according to histological subtypes, and patients were further subdivided into low- and high-expression groups based on the median relative PPM1B expression of the entire cohort. Kaplan–Meier survival analysis of OS revealed a significant association between tumor type (SCC vs. ADC) and PPM1B relative expression with *p* = 0.009 (Figure 1D), while ADC patients with high PPM1B expression presented the longest median OS (45 months), followed by the ADC low PPM1B expression group (37 months). Significant difference in survival outcome with *p* = 0.04 was confirmed between SCC and ADC groups representing high PPM1B expression. Furthermore, both SCC subgroups exhibited poorer survival outcomes, with a median OS of 17 months in the low expression group and 10.5 months in the high expression group.

### 3.2. The Expression of the MYPT1 Regulatory Subunit of MP Is Elevated in ADC and SCC Tissues

**Figure 2:**
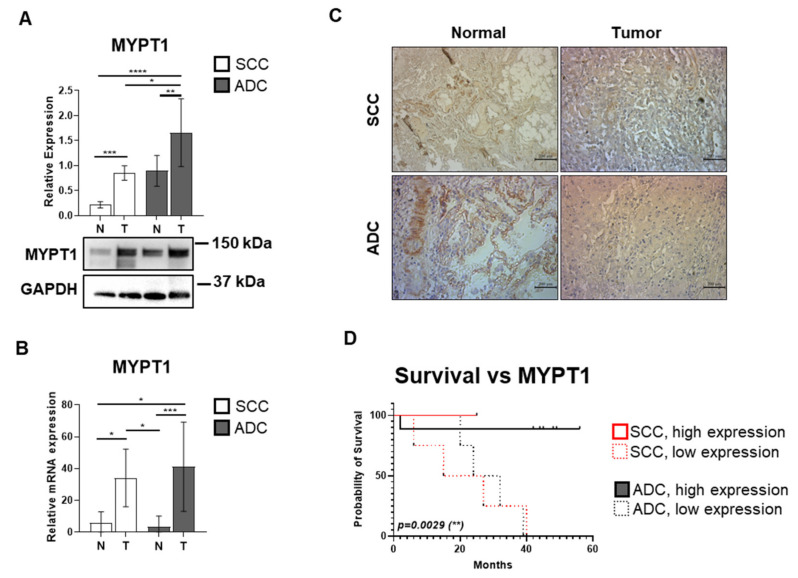
MYPT1 expression and its correlation with survival outcomes in squamous cell carcinoma (SCC) and adenocarcinoma (ADC) lung cancer tissues. (**A**) Protein extracts from SCC and ADC tumor samples, along with their matched non-cancerous lung controls, were subjected to SDS-PAGE and analyzed by Western blot using anti-MYPT1 antibody. GAPDH was used as a loading control. Results are illustrated as bar charts representing the mean ± SD for each group (*n* = 8–19). The original WB images are shown in Figure S4. (**B**) MYPT1 mRNA expressions were quantified by RT-PCR in both tumor and control tissues of ADC and SCC origin. Cp values were normalized against the housekeeping genes GAPDH and Cyclophilin A, and relative expression levels were plotted as a bar chart, where one column represents mean ± SD (*n* = 3–10 per group). Group comparisons were made using one-way or two-way ANOVA followed by Tukey’s post hoc test, and two-group comparisons employed unpaired two-tailed *t*-tests. Statistical significance was defined as *p* < 0.05 (*), *p* < 0.01 (**), *p* < 0.001 (***), and *p* < 0.0001 (****). (**C**) Representative images of MYPT1 immunohistochemical staining (DAB) are shown for tumor and control tissues from SCC and ADC patients. Scale bar: 200 µm. (**D**) Kaplan–Meier survival curves illustrating the overall survival of patients with SCC and ADC, stratified according to MYPT1 expression levels. Time is expressed in months since study inclusion. Patients were grouped by histological subtype (SCC in red, ADC in black), and within each group, high and low MYPT1 expression cohorts were defined based on the cohort-wide median expression value. Statistical evaluation of survival differences was performed using the log-rank (Mantel–Cox) test. Asterisks (**) denote statistically significant differences (*p* < 0.01). The log-rank test statistic approximates the Chi-square distribution.

The next investigated element of the PPM1B/MP/PRMT5/histone oncogenic pathway is MYPT1, the regulatory subunit of MP. MP is known to have a tumor suppressor role by dephosphorylating the activating phospho-Thr80 site of PRMT5, a mechanism previously demonstrated in hepatocellular carcinoma cells [28]. We aimed to investigate whether ADC and SCC lung cancers are characterized by this regulatory mechanism. MYPT1 expression was increased in both SCC and ADC tumor samples, by 3.93-fold (*p* = 0.0002) and 1.96-fold (*p* = 0.003), respectively, compared to their control tissue counterparts (Figure 2A). Also, SCC tumor samples represented a 0.49-fold (*p* = 0.026) decrease in MYPT1 expression compared to cancerous tissue derived from ADC patients. Quantitative RT-PCR measuring *MYPT1* mRNA expression (Figure 2B) showed a 12.111-fold (*p* = 0.0008) and 5.68-fold (*p* = 0.033) increase in ADC and SCC tumor tissues compared to their matched controls, respectively. Also, in ADC tumor tissues, *MYPT1* mRNA expression was 6.86-fold higher compared to SCC patient control tissues (*p* = 0.016). In SCC tumor tissues, MYPT1 mRNA expression was also 10.03-fold higher compared to ADC patient control tissues (*p* = 0.05). Figure 2C shows representative IHC images of SCC, ADC, and normal tissues, all probed for MYPT1. The DAB signal intensity appears similarly strong in both control tissue groups. In the tumor tissues, MYPT1 staining is also strong, with the signal in ADC tissues potentially appearing more intense than in SCC tissues, suggesting a higher MYPT1 protein expression in ADC. 

Kaplan–Meier survival analysis was performed to evaluate the association between MYPT1 expression and OS (Figure 2D). The SCC and ADC patient groups were classified into high and low MYPT1 expression subgroups, based on the median relative expression level across the entire cohort. Log rank test revealed a significant correlation between survival and MYPT1 expression (*p* = 0.003), as ADC high MYPT1 expressing group ranking highest with median OS of 45 months, followed by 28 months of OS in ADC low MYPT1 expressing group. In contrast, within the SCC group, the low-expression and the high-expression subgroups had a median OS of 21 and 25 months, respectively (*p* = 0.006).

### 3.3. The Inhibitory MYPT1 Thr853 Phosphorylation Increases Significantly in Lung Cancer

**Figure 3:**
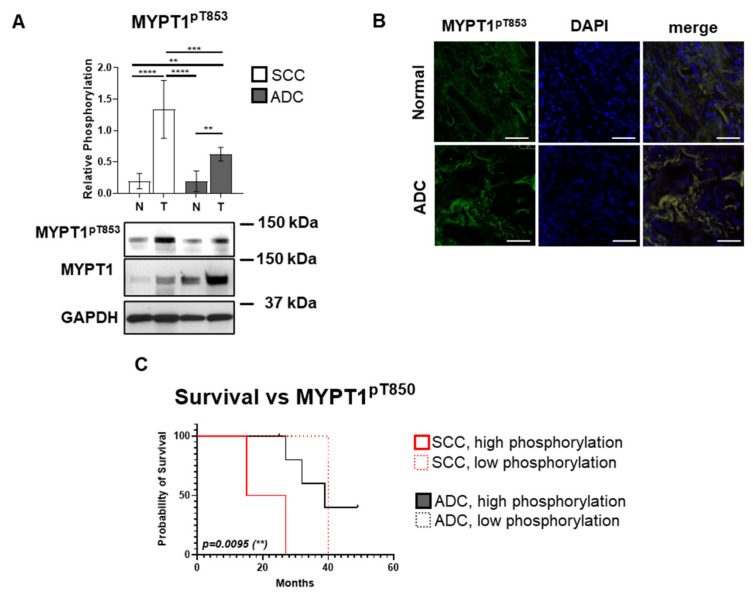
MYPT1 phosphorylation and its association with survival outcome in squamous cell carcinoma (SCC) and adenocarcinoma (ADC) lung cancer tissues. (**A**) Protein lysates from SCC and ADC tumors and their corresponding non-malignant lung tissues were analyzed by Western blot with anti-MYPT1^pT853^ antibody. Phosphorylation levels were normalized to total MYPT1 expression. Bar chart displays relative phosphorylation where one bar represents mean ± SD (*n* = 3–18). Statistical analysis was performed using one-way or two-way ANOVA with Tukey’s multiple comparisons test for multiple groups, and unpaired two-tailed *t*-tests for pairwise comparisons. Significance levels are indicated as *p* < 0.01 (**), *p* < 0.001 (***), and *p* < 0.0001 (****). The original WB images are shown in Figure S5. (**B**) Immunofluorescent labeling of MYPT1^pT853^ (green, Alexa Fluor 488) in tumor and control tissues. Nuclear counterstaining was performed using DAPI (blue). Scale bar: 100 µm. (**C**) Kaplan–Meier survival analysis of SCC and ADC patients, stratified by MYPT1^pT853^ phosphorylation levels. Survival is plotted as a function of time (months) passed from enrollment in the study. Patients were categorized by histological subtype (SCC: red; ADC: black), and further subdivided into low and high phosphorylation groups using the median phosphorylation value of the entire cohort as a threshold. Survival curves were statistically compared using the log-rank (Mantel–Cox) test. Statistically significant differences (*p* < 0.001) are indicated by asterisks (**). The test statistic approximates the Chi-square distribution.

Elevated phosphorylation of MYPT1 at Thr853 residue indicates a decrease in MP holoenzyme activity [27,39], which results in the decreased dephosphorylation of the activating phosphorylation at the Thr80 site of PRMT5 [28]. As in the previous analyses, we evaluated the relative phosphorylation level of MYPT1 at Thr853 (MYPT1^pT853^) (Figure 3A). Our results indicate that MYPT1 Thr853 phosphorylation was significantly elevated in SCC tumor tissues, with a 6.89-fold increase compared to matched normal lung tissues (*p* < 0.0001), and a 6.98-fold increase compared to the control tissues of ADC patients (*p* < 0.0001). Similarly, ADC tumor tissues exhibited a 3.25-fold increase in MYPT1^pT853^ relative phosphorylation compared to their corresponding control tissues (*p* = 0.0055), which shows a clear correlation between the phosphorylation at the inhibitory site and tumor formation. Furthermore, comparing ADC and SCC tumor samples, MYPT1^pT853^ phosphorylation was more pronounced in SCC tumors compared to ADC, by a 2.147-fold increase (*p* = 0.0004). Immunofluorescent labeling of MYPT1^pT853^ in tumor and control tissues shows that the MYPT1^pT853^ signal is elevated throughout the cancer cells (ADC) and overlaps with the nuclear DAPI staining (blue), indicating that MYPT1^pT853^ localizes in the cell nucleus as well in lung cancer tissue (Figure 3B).

Figure 3C presents a Kaplan–Meier correlation between OS and relative phosphorylation levels of MYPT1^pT853^ in ADC and SCC patients (*p* = 0.0095). ADC patients had a median OS of 39 months in the high-phosphorylation and 45 months in the low-phosphorylation subgroups, respectively. The OS of SCC patients reached 21 months in the high phosphorylation and 25 months in the low phosphorylation subgroups. Log-rank test comparing SCC and ADC high phosphorylation groups revealed a difference (*p* = 0.0344) in survival outcome, dependent on MYPT1 phosphorylation in correspondence to histological subtype, which shows a clear correlation between increased survival, decreased MYPT1 phosphorylation, and increased MP activity.

### 3.4. SCC and ADC Tissue Samples Exhibit Elevated PRMT5 Expression Levels

**Figure 4:**
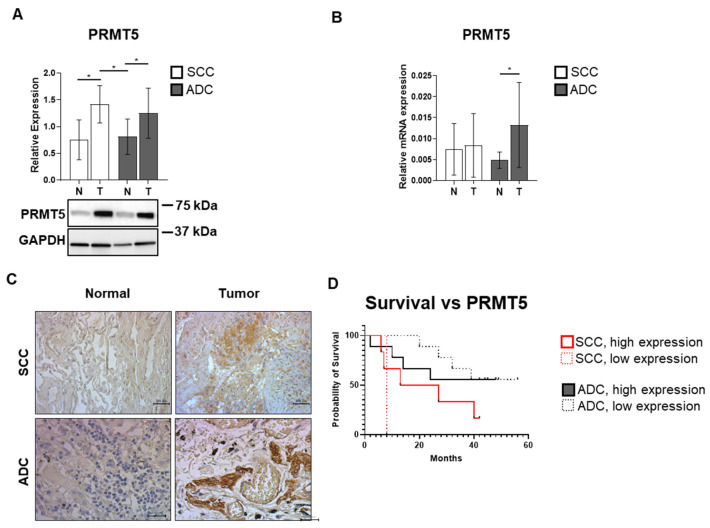
PRMT5 expression and its relationship to survival outcomes in lung adenocarcinoma (ADC) and squamous cell carcinoma (SCC). (**A**) Total protein lysates from ADC and SCC tumor tissues, along with their respective non-tumorous controls, were analyzed by SDS-PAGE and Western blotting using an anti-PRMT5 antibody. GAPDH served as a loading control. Quantified protein levels are presented as bar graphs showing mean ± SD for each group (*n* = 5–16). The original WB images are shown in Figure S6. (**B**) PRMT5 mRNA expression in both tumor and matched control tissues from ADC and SCC samples was determined by RT-PCR. Cp values were normalized to GAPDH and Cyclophilin A housekeeping genes, and relative mRNA expression is shown as a bar graph (mean ± SD, *n* = 3–8). Statistical comparisons across multiple groups were performed using one-way or two-way ANOVA followed by Tukey’s multiple comparisons test; pairwise comparisons were assessed using unpaired two-tailed *t*-tests, where *p* < 0.05 (*). (**C**) Representative images of immunohistochemical staining (DAB) for PRMT5 are shown in tumor and control tissues from SCC and ADC patients. Scale bar: 200 µm. (**D**) Kaplan–Meier curves displaying overall survival stratified by PRMT5 expression in patients with ADC and SCC. Time is shown in months from the date of study entry. Patients were grouped based on histological subtype (SCC: red; ADC: black) and further classified into low and high PRMT5 expression according to the median expression value across the total cohort.

To assess PRMT5 expression in ADC and SCC tissues, protein lysates were analyzed (Figure 4A). Both ADC and SCC tumor samples displayed significantly increased PRMT5 protein expression compared to their corresponding controls, with fold changes of 1.54 (*p* = 0.02) and 1.88 (*p* = 0.042), respectively. To corroborate these findings, *PRMT5* mRNA levels were quantified using RT-qPCR (Figure 4B). A 2.71-fold (*p* = 0.04) upregulation was observed in ADC tumor tissues but not in SCC samples. To further characterize alterations of PRMT5 expression under tumorigenic conditions, we performed DAB immunohistochemistry on ADC and SCC tissue sections (Figure 4C). A markedly stronger DAB reaction was evident in ADC tumor tissue slides. Kaplan–Meier survival analysis did not reveal a statistically significant association between PRMT5 expression levels (high vs. low) and overall survival in either ADC or SCC lung cancer patients (Figure 4D).

### 3.5. Activating PRMT5^pT80^ Phosphorylation Is Enhanced in SCC and ADC Lung Cancer

**Figure 5:**
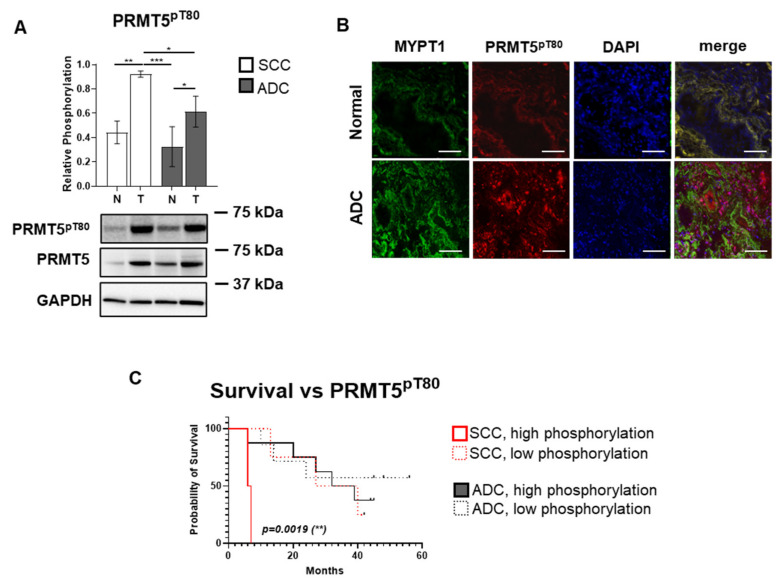
PRMT5 Thr80 phosphorylation and its link to survival in lung squamous cell carcinoma (SCC) and adenocarcinoma (ADC) tissues. (**A**) Protein extracts from SCC and ADC tumor samples and their matched non-tumorous lung tissues were subjected to Western blot analysis using anti-PRMT5^pT80^ antibody. Phosphorylation intensity was normalized to total PRMT5 expression. Quantitative data are presented as bar charts, where each bar reflects the mean ± SD (*n* = 3–15 per group). Group differences were assessed using one-way or two-way ANOVA followed by Tukey’s post hoc test for multiple comparisons and unpaired two-tailed *t*-tests for pairwise analyses. Statistical significance is indicated as *p* < 0.05 (*), *p* < 0.01 (**), and *p* < 0.001 (***). The original WB images are shown in Figure S7. (**B**) Immunofluorescence staining of MYPT1 (green, Alexa Fluor 488) and PRMT5^pT80^ (red, Alexa Fluor 546) was performed on tumor and control samples. Nuclei were counterstained with DAPI (blue). Scale bar: 100 µm. (**C**) Kaplan–Meier survival curves showing overall survival of SCC and ADC patients in relation to PRMT5^pT80^ phosphorylation. Time is expressed in months following study entry. Patients were stratified by histological subtype (SCC: red; ADC: black), and within each group, further categorized into high or low phosphorylation groups based on the median phosphorylation value of the entire cohort. Survival distributions were compared using the log-rank (Mantel–Cox) test. Statistically significant differences (*p* < 0.01) are marked with double asterisks (**). The test statistics follow an approximate Chi-square distribution.

To quantify the phosphorylation at the PRMT5^T80^ residue, Western blot and immunofluorescent staining were applied. Relative to corresponding controls, SCC and ADC tumor tissues demonstrated significantly elevated phosphorylation levels compared to their healthy counterparts, with a 2.08-fold (*p* = 0.003) and a 1.89-fold (*p* = 0.013) increase, respectively (Figure 5A). Furthermore, phosphorylation levels were 1.5-fold (*p* = 0.03) higher in SCC compared to ADC tumor samples. Immunofluorescent staining further confirmed the elevated phosphorylation at Thr80 of PRMT5 in tumor tissues (Figure 5B). In merged images, co-localization (purple) of PRMT5^pT80^ (red) and DAPI (blue) staining, indicating pronounced nuclear localization and activating phosphorylation of PRMT5. Kaplan–Meier survival analysis revealed a significant association between tumor type (SCC vs. ADC), PRMT5^pT80^ phosphorylation level (high vs. low), and patient survival (*p* = 0.002, Figure 5C). Specifically, SCC patients with high phosphorylation exhibited a median survival of 6.5 months, compared to 33.5 months with low phosphorylation. ADC patients showed median survivals of 35.5 and 45 months for high and low phosphorylation groups, respectively. Log-rank test comparing SCC and ADC patients with high PRMT5^pT80^ phosphorylation revealed a significant difference between OS (*p*= 0.019), while a difference in survival (*p* = 0.018) was observed between SCC high and low PRMT5^pT80^ phosphorylation groups, which clearly shows the correlation between PRMT5 activity and poor survival.

### 3.6. Symmetrical Dimethylation of H2A Protein Occurs in SCC and ADC Without Changes in Its Protein Expression

**Figure 6:**
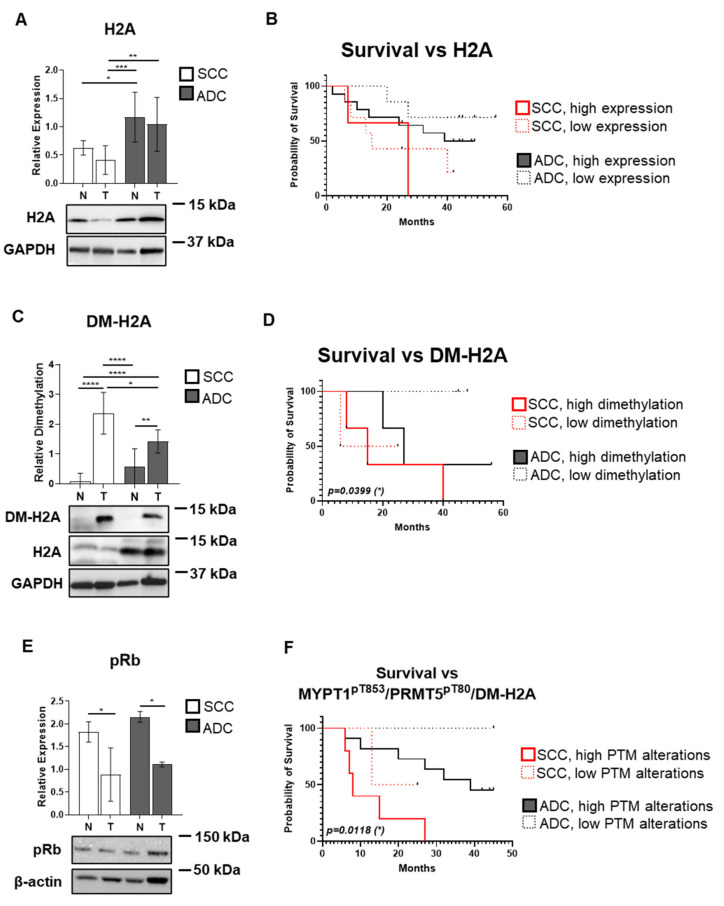
H2A expression, symmetric dimethylation, and their associations with retinoblastoma protein expression and survival in lung adenocarcinoma (ADC) and squamous cell carcinoma (SCC). (**A**,**C**) Protein lysates from tumor and matched control lung tissues of SCC and ADC origin were analyzed by Western blot using anti-H2A (**A**) and anti-symmetrically dimethylated H2A (DM-H2A) (**C**) and anti-retinoblastoma protein (pRb) (**E**) antibodies. The original WB images are shown in Figures S8–S10. Total H2A levels were normalized to GAPDH, dimethyl-H2A signal was normalized to total protein levels (**C**), and the pRb expression was normalized to β-actin (**E**). Quantitative data are displayed as bar graphs showing mean ± SD (*n* = 3–20 per group). Group comparisons were evaluated using one-way or two-way ANOVA followed by Tukey’s post hoc analysis, and pairwise comparisons were assessed using unpaired two-tailed *t*-tests. Significance thresholds were set at *p* < 0.05 (*), *p* < 0.01 (**), *p* < 0.001 (***), and *p* < 0.0001 (****). (**B**,**D**,**F**) Kaplan–Meier curves depict overall survival of lung cancer patients stratified by H2A expression (**B**), H2A symmetric dimethylation (**D**), and the post-translational modifications (PTM) of MYPT1^pT853^, PRMT5^pT80^ phosphorylation, and H2A dimethylation levels (**F**). Time is represented as months from study inclusion. Patients were grouped according to histological subtype (SCC: red; ADC: black), and further divided into low and high expression and dimethylation and PTM subgroups using the median value of the entire dataset as a cutoff. Differences between survival curves were tested using the log-rank (Mantel–Cox) method. Statistically significant associations (*p* < 0.05) are indicated by an asterisk (*). The log-rank test statistic approximates a Chi-square distribution.

We examined the expression and symmetric dimethylation (DM) of the terminal effector of the PPM1B/MP/PRMT5/H2A signaling axis, the H2A protein. The highest expression of H2A was observed in control samples. Significantly, 2.54-fold (*p* = 0.005) elevation of H2A expression was observed in ADC compared to SCC samples (Figure 6A). Kaplan–Meier analysis did not indicate significant correlations with H2A expression either in SCC or ADC (Figure 6B). Symmetric DM of H2A showed a 24.74-fold (*p* < 0.0001) and a 2.49-fold (*p* = 0.0015) elevation in SCC and ADC tumor samples compared to their controls, respectively (Figure 6C). We have also supported our results with the expression and symmetric demethylation of histone H4, showing the same results as H2A (Figure S2A,B). Additionally, SCC tumors demonstrated significantly higher DM levels compared to ADC tumor samples by 1.66-fold (*p* = 0.017). Kaplan–Meier analysis indicated that the level of symmetric DM of H2A proteins associates significantly with the survival (*p* = 0.04) (Figure 6D). For SCC patients, the median survival was 15.5 months (low DM) versus 15 months (high DM). For ADC patients, these values were 45 and 27 months, respectively. To verify the effect of histone modification on gene expression and the effects of the PPM1B/MP/PRMT5/H2A proto-oncogenic axis on target gene regulation, we examined changes in the expression levels of the tumor suppressor retinoblastoma protein (pRb) (Figure 6E). The pRb protein expression was assessed in SCC and ADC patient tumor samples compared to their respective controls by Western blot analysis. pRb protein levels showed a marked decrease in SCC tumor samples, with a 2.06-fold reduction (*p* = 0.011) compared to controls. Similarly, ADC tumor samples exhibited a 1.94-fold decrease (*p* = 0.0145), indicating a consistent downregulation of pRb expression in tumors, likely reflecting the downstream impact of the dysregulated PPM1B/MP/PRMT5/H2A pathway. To further illustrate the relevance of the PPM1B–MYPT1–PRMT5–H2A proto-oncogenic pathway dysfunction, an aggregated Kaplan–Meier survival analysis was performed to compare survival differences among patient subgroups (Figure 6F). Patients were first stratified by histological subtype (ADC vs. SCC) and further subdivided based on consistently low or inconsistently high levels of PTMs of MYPT1^pT853^ and PRMT5^pT80^ phosphorylation, as well as H2A symmetric dimethylation. The log-rank test revealed a significant difference in overall survival between subgroups (*p* = 0.01). Adenocarcinoma patients with low post-translational modification levels showed the most favorable prognosis (median survival of 45 months), whereas SCC patients with high PTM levels exhibited the poorest survival (median survival of 15 months).

## 4. Discussion

This study provides the first comprehensive analysis of the PPM1B/MP/PRMT5/histone signaling pathway in human lung cancer tissues, revealing a novel oncogenic mechanism that distinguishes between ADC and SCC subtypes. Our findings demonstrate that dysregulation of this pathway contributes to lung tumorigenesis through epigenetic modifications that ultimately suppress tumor suppressor gene expression.

Our results establish PPM1B as a potential tumor suppressor in lung cancer, with significantly reduced expression observed in both SCC and ADC tissues compared to normal lung tissue, with a more pronounced, 0.31-fold (*p* = 0.029) decrease in SCC (Figure 1A). This reduction correlates with poorer overall survival, particularly in SCC patients (Figure 1D). This finding is consistent with emerging evidence suggesting that PPM1B acts as a tumor suppressor in gastric adenocarcinoma [40] or breast cancer [41,42]. The decreased PPM1B expression observed in our study aligns with previous reports in cervical carcinoma, where PPM1B downregulation was associated with increased cellular proliferation and transformation [29,43]. Interestingly, while protein expression was consistently reduced in both histological subtypes, mRNA levels showed no significant differences (Figure 1B). This discrepancy between mRNA and protein expression has been reported in other cancer types [40,44] and may indicate the involvement of microRNA regulation [45], protein degradation pathways [44], or translational control mechanisms [46]. This is all in line with findings that pleckstrin-2-promoted PPM1B degradation plays an important role in transforming growth factor-β-induced breast cancer cell invasion and metastasis [42]. The survival analysis revealed that PPM1B expression levels have differential prognostic implications depending on tumor histology (Figure 1D). In adenocarcinoma patients, higher PPM1B expression was associated with better overall survival (45 months versus 37 months), supporting its tumor suppressor function. However, the prognostic value in SCC patients was less pronounced, suggesting that other molecular mechanisms may predominate in this histological subtype. Data also showed a significant difference in overall survival of ADC and SCC patients represented with high PPM1B expression (*p* = 0.04) (Figure 1D).

Contrary to our initial hypothesis based on previous hepatocellular carcinoma studies, MYPT1 protein and mRNA expression were significantly elevated in both SCC and ADC tumor tissues compared to normal controls (Figure 2A,B). This unexpected finding suggests that the regulatory mechanisms governing MP activity in lung cancer may differ from those in HCC. The dramatic increase in MYPT1 mRNA expression (12-fold in ADC and 5.68-fold in SCC) indicates robust transcriptional activation, possibly as a compensatory response to increased oncogenic signaling. PPM1B regulates MP activity by dephosphorylating MYPT1 at its inhibitory Thr853 and Thr696 sites [29]. Loss of PPM1B thus leads to sustained MYPT1 phosphorylation, reduced MP activity, and enhanced PRMT5 activation—a cascade that promotes tumorigenesis via epigenetic silencing of tumor suppressor genes. Our findings align with previous studies in hepatocellular carcinoma, where MYPT1 was shown to act as a tumor suppressor by regulating PRMT5 activity [28]. Despite the increased MYPT1 expression, the significantly elevated phosphorylation at Thr853 suggests that the protein is functionally inactivated (Figure 3A). This phosphorylation event, catalyzed by ROK and other kinases, prevents MYPT1 from effectively dephosphorylating its substrates, including PRMT5. The paradoxical increase in MYPT1 expression coupled with its functional inactivation through phosphorylation represents a sophisticated mechanism by which cancer cells can overcome tumor suppressor pathways while maintaining the appearance of normal regulatory protein levels.

The survival analysis revealed complex relationships between MYPT1 expression and phosphorylation and patient outcomes (Figure 2D and Figure 3C). Both SCC and ADC low phosphorylation MYPT1 groups showed significantly longer survival outcomes (25 and 45 months, respectively), compared to high phosphorylation countergroups (21 and 39 months), suggesting that total MYPT1 levels may not be as prognostically relevant as its phosphorylation status. In contrast, ADC patients with low MYPT1 phosphorylation showed remarkably better survival (45 months versus 39 months), indicating that this histological subtype may confer a survival advantage. The difference in overall survival between SCC and ADC patients presented with high MYPT1^pT853^ phosphorylation was also perceivable (*p* = 0.034).

These data support previous findings that the unbalanced and constant phosphorylation of MYPT1 at Thr853 was suggested to be related to cancer formation and mutations, as well as altered phosphorylation and expression of MYPT1 were documented in various cancer types [47,48]. Interestingly, decreased expression of MYPT1 did not always accompany tumor development, but increased phosphorylation of MYPT at Thr853 did. This phenomenon may be explained, at least in part, by the regulation of MP with PPM1B.

Our findings confirm that PRMT5 plays a central oncogenic role in lung cancer through both increased expression and enhanced activating phosphorylation at Thr80. The elevated PRMT5 expression observed in both SCC and ADC tissues is consistent with previous reports demonstrating PRMT5 overexpression in various cancer types [20,21,22,23]. More importantly, the significant increase in Thr80 phosphorylation in tumor tissues (Figure 5A) indicates enhanced enzymatic activity, as this phosphorylation event is known to increase PRMT5 methyltransferase activity. The clinical significance of PRMT5^pT80^ phosphorylation is underscored by its strong association with patient survival (Figure 5C). SCC patients with high PRMT5^pT80^phosphorylation showed dramatically reduced median survival (6.5 months versus 33.5 months), highlighting this modification as a potential prognostic biomarker and therapeutic target. The less pronounced but still significant survival differences in ADC patients (35.5 versus 45 months) suggest that PRMT5 activation may be particularly critical in the more aggressive SCC subtype. This hypothesis is also supported by the observed difference in overall survival between SCC and ADC patients presented with high PRMT5^pT80^ phosphorylation. The nuclear localization of both total and phosphorylated PRMT5 observed in our immunofluorescence studies (Figure 5B) confirms its role in chromatin modification. This subcellular distribution is consistent with the function of PRMT5 as a histone methyltransferase and supports its involvement in epigenetic gene regulation. The enhanced nuclear accumulation in tumor tissues further supports the oncogenic activation of this pathway.

The downstream effect of PRMT5 activation—symmetric dimethylation of histone H2A—was significantly elevated in both tumor types (Figure 6C). While total H2A expression was observed to be the highest in ADC control tissues (Figure 6A), increased dimethylation correlated with reduced survival in ADC patients, highlighting its potential as a prognostic marker. Histone H2A symmetric dimethylation as a functional readout showed a dramatic increase in tumor tissues and provides direct evidence of enhanced PRMT5 enzymatic activity in vivo. The particularly striking 24.74-fold increase in SCC and 2.49-fold increase in ADC tissues demonstrate that the upstream pathway alterations we identified translate into functional downstream consequences. This histone modification is known to be associated with gene silencing, particularly of tumor suppressor genes, providing a mechanistic link between PRMT5 activation and oncogenesis. The differential magnitude of H2A (Figure 6A,B) and H4 (Figure S2) dimethylation between histological subtypes is intriguing and may reflect distinct molecular characteristics of these tumor types. The higher fold-change observed in SCC compared to ADC suggests that squamocellular carcinomas may be more dependent on epigenetic silencing mechanisms, while ADC may rely more heavily on other oncogenic pathways. The lower expression of pRb tumor suppressor in ADC and SCC samples (Figure 6E) provides indirect evidence for histone symmetric dimethylation, thus confirming the MP/PRMT5 pathway-dependent effect of gene expression inhibition already described by Sipos, A. et al. [24] in hepatocellular carcinoma. This observation is supported by the survival analysis, where H2A dimethylation levels showed stronger prognostic significance in SCC compared to ADC patients .

Our study elucidates a novel oncogenic signaling axis in lung cancer and highlights the differential molecular landscape between SCC and ADC. These findings also open new avenues for biomarker development and therapeutic intervention. Importantly, our study identifies PPM1B as a potential tumor suppressor and prognostic biomarker in lung cancer. Its downregulation appears to initiate a cascade of molecular events that culminate in epigenetic silencing and tumor progression that support targeting the restoration of its function or mimicking its tumor suppressor activity. In addition, several PRMT5 inhibitors are currently in clinical trials [21,22,23,49], and our identification of phosphorylation at PRMT5^pT80^ as a strong prognostic biomarker, particularly in SCC, provides a potential tool for risk stratification and treatment selection. Furthermore, Hu et al. found in their study that inhibition of PRMT5 by shRNA or pharmacological approaches attenuated tumor progression and caused enhanced PD-L1 expression and improved response to immune checkpoint inhibitors [50]. This suggests that the most modern lung cancer therapy—the immune checkpoint inhibition of PD-L1—can be enhanced with PRMT5 inhibitors, supporting the development of PRMT5-targeted therapies and the application of combined therapies. Additionally, targeting the kinases responsible for MYPT1^pT853^ and PRMT5^pT80^ phosphorylation could provide alternative therapeutic approaches to disrupting this oncogenic pathway and may trigger personalized treatment approaches. However, the limitations of the study are the relatively small sample size, which, although it shows a clear correlation with post-translational modification of elements of the oncogenic signaling pathway, is primarily of diagnostic significance.

## 5. Conclusions

In conclusion, our study reveals that the PPM1B/MP/PRMT5/histone oncogenic signaling pathway plays a role in lung cancer development and progression. The dysregulation of this pathway, characterized by decreased PPM1B expression, functional inactivation of MYPT1 despite increased expression, enhanced PRMT5 activity, and consequent increase in histone H2A methylation, provides new insights into the molecular mechanisms underlying lung tumorigenesis. The differential patterns observed between ADC and SCC subtypes highlight the molecular heterogeneity of lung cancer and support histology-specific therapeutic approaches.

## Data Availability

The data generated and analyzed during this study include sensitive patient information and are therefore not publicly available due to ethical and privacy restrictions. Access to anonymized data may be granted by the corresponding author upon reasonable request and with appropriate ethical approval.

## Supplementary Materials

The following supporting information can be downloaded at https://www.mdpi.com/article/10.3390/biom15111581/s1, Table S1. Primers; Table S2. Antibodies; Table S3. Distribution of histological types; Table S4. Distribution of pathological stages; Figure S1. Overview of lung cancer tissue samples; Figure S2. H4 symmetric dimethylation is downregulated due to the inavtivation of the PPM1B/MP/PRMT5 protooncogenic axis in lung adenocarcinoma (ADC) and squamous cell carcinoma (SCC); Figure S3. The original WB images of Figure 1A; Figure S4. The original WB images of Figure 2A; Figure S5. The original WB images of Figure 3A; Figure S6. The original WB images of Figure 4A; Figure S7. The original WB images of Figure 5A; Figure S8. the original WB images of Figure 6A; Figure S9. The original WB images of Figure 6C; Figure S10. The original WB images of Figure 6E; Figure S11. The original WB images of Figure S2A,B. References cited in Supplementary Materials [9,31,32,37,38].

## Abbreviations

ADC	Adenocarcinoma
ANOVA	Analysis of Variance
BSA	Bovine Serum Albumin
DAB	3,3′-Diaminobenzidine
DAPI	4′,6-Diamidino-2-phenylindole
DFS	Disease-Free Survival
DM	Dimethylation
EDTA	Ethylenediaminetetraacetic Acid
GAPDH	Glyceraldehyde 3-Phosphate Dehydrogenase
GDPR	General Data Protection Regulation
H2A	Histone H2A
HCC	Hepatocellular Carcinoma
IHC	Immunohistochemistry
MP	Myosin Phosphatase
MYPT1	Myosin Phosphatase Targeting Subunit 1 (regulatory subunit of MP)
OS	Overall Survival
PP1c	Protein Phosphatase 1 catalytic subunit
PPM1B	Mg^2+/^Mn^2+^-dependent Protein Phosphatase 1B
pRb	retinoblastoma protein
PRMT5	Protein Arginine Methyltransferase 5
RT-qPCR	Reverse Transcription-quantitative Polymerase Chain Reaction
SCC	Squamous Cell Carcinoma
SD	Standard Deviation
SDS	Sodium Dodecyl-Sulfate
SCLC	Small Cell Lung Cancer
TBST	Tris-Buffered Saline with Tween 20
