# Supplementary material for: Decreased PPM1B Expression Drives PRMT5-Mediated Histone Modification in Lung Cancer Progression
Source: Biomolecules. 2025 Nov 11;15(11):1581. doi: 10.3390/biom15111581 (PMC12649863; doi:10.3390/biom15111581)
Supplement: Supplementary file 1 [file biomolecules-15-01581-s001.zip › biomolecules-3959801-supplementary.pdf]

Supplementary Materials

Supplementary results

Patients

A total of 38 patient samples were collected in this study, and their clinical data were analyzed. Among these 38 patients, 12 were female (33%) and 26 were male (67%). The average age of the female patients was 63.92 years, while that of the male patients was 65.81 years. The average smoking history, measured in pack-years, was 26.42. Only 2 patients were non-smokers. From an oncological perspective, the following treatments had been administered prior to our study. Three patients had a history of previous tumors that had been surgically removed: One patient had undergone surgery for breast carcinoma (the current histology at our institution showed lung adenocarcinoma), another one patient had been operated on for colon adenocarcinoma (the current histology showed a colon carcinoma metastasis) and one patient had undergone surgery for colon carcinoma (the current histology at our institution revealed lung adenocarcinoma). In addition, neoadjuvant chemotherapy was administered to three patients: one adenocarcionoma and 2 squamous cell carcionoma patients. In all enrolled patients, the first confirmed or suspected lung lesion was identified at the time of inclusion, and none had a prior history of lung malignancy; thus, none of the cases represented recurrent lung cancer.

Classification was performed according to the 2015 WHO criteria [9]. As expected, the more common histological types were adenocarcinoma (21 patients, 55.26%) and squamous cell carcinoma was 10 patients, 26.31%). In cases where no biopsy was performed prior to surgery to confirm tumor suspicion, several non-NSCLC (non-small cell lung cancer) lesions were identified postoperatively. These cases were excluded from further analysis. The excluded histological types (one case each) were: gastric cancer metastasis, sarcoidosis, colon cancer metastasis, necrotizing granulomatous inflammation (non-tuberculous), neuroendocrine carcinoma, large cell carcinoma,

and small cell carcinoma. (Table S3) During data collection, pathological stages were also determined, and the stages of the NSCLC tumors were analyzed (Table S4). Stage classification was based on the 8th edition of the TNM (tumor, node, and metastasis) staging system [51]. The distribution of TNM stages is shown in Fig. S1.A. Experimental results were also correlated with tumor stage. Among the T stages, T2 tumors were the most common (17 patients – 54.84%), followed by T1 tumors (10 patients – 32.26%), and T3 tumors were the least frequent (4 patients – 12.91%). The majority of patients did not have lymph node metastases, with 19 patients (61.29%) classified as N0. Six patients (19.35%) had lymph node metastases in the ipsilateral hilar region (N1), and another six patients (19.35%) had mediastinal lymph node metastases (N2). Most patients in the N2 group also had T2 tumors (Fig. 1B). The tumor grades were distributed as follows: G1 in 3 patients (8.82%), G2 in 18 patients (52.94%), and G3 in 13 patients (34.21%). In the examined population, few tumors harbored druggable driver mutations. Specifically, ALK mutations were detected in 3 cases, and an EGFR mutation (exon 19 deletion) was found in 1 case. Overall survival (OS) is considered the "gold standard" endpoint in clinical trials [37]. For patients who undergo R0 resection, disease-free survival (DFS) is also a critical measure [52]. After data collection, OS and DFS were calculated (Fig. S1.C and 1.D). OS data were further correlated with other factors, such as protein expression levels. The median OS was 44 months for adenocarcinoma patients and 20 months for squamous cell carcinoma patients ($p = 0.0170$). The median DFS was 17 months in the adenocarcinoma group and 14 months in the squamous cell carcinoma group ($p = 0.0326$). Both OS and DFS were significantly better among adenocarcinoma patients. During the data collection period, of all 38 patients, 13 patients (34.21%) died, 15 patients (39.47%) were alive and tumor-free, and 10 patients (26.31%) were alive but experienced tumor recurrence. We analyzed tumor location by lung lobe. Most malignant tumors were found in the

upper lobes, predominantly in the right upper lobe [53]. The most frequent site was the right upper lobe, with tumors in 12 patients (39%). In 8 patients (26%), tumors were located in the left upper lobe; in 1 patient (3%), the tumor was in the right middle lobe; and in both lower lobes, 5 patients (16%) had tumors (Fig. S1.B).

To further elucidate the effects on downstream elements in the presence of downregulation of the PPM1B/MP/PRMT5 proto-oncogenic axis, tumor and corresponding control tissue lysates from SCC and ADC patients were subjected to Western blot analysis (Figure S2). According to our findings, total H4 expression was not altered (Figure S2A); however, symmetric dimethylation of H4 was upregulated in SCC tumor samples by approximately 2.55-fold ($p = 0.05$) compared to the control (Figure S2B) l. Similarly, comparison of ADC tumor and control tissues revealed an approximately 2.50-fold ($p = 0.0325$) upregulation of H4 symmetric dimethylation in tumor samples relative to the corresponding controls (Figure S2B). These findings indicate a widespread downstream effect associated with downregulation of the PPM1B/MP/PRMT5 proto-oncogenic axis.

Supplementary Tables

Table S1. Primers

Gene	Directio n	Seqence 5'->3'	Concentratio n per reaction	Tm
Human GAPDH	Forward	*AGCCTCAAGATCAGCAATG*	0,4 µM	52,6°C
Human GAPDH	Reverse	*ATGGACTGTGGTCATGAGTCCTT*	0,4 µM	57,9°C
Human Cyclophillin A	Forward	*GTCTCCTTTGAGCTGTTTGCAGAC*	0,4 µM	58,2°C
Human Cyclophillin A	Reverse	*CTTGCCACCAGTGCCATTATG*	0,4 µM	56,7°C
Human MYPT1	Forward	*CAACAACCCTGACTACAACTAC*	0,4 µM	60,0°C
Human MYPT1	Reverse	*TCTCCTTCTTTCTCCTCTTCTCT*	0,4 µM	60,7°C
Human PRMT5	Forward	*CGGAGAAGGGCAGACTA*	0,4 µM	52.4°C
Human PRMT5	Reverse	*CAATTTCAAGAGCCACTGC*	0,4 µM	58.6°C
Human PPM1B	Forward	*GACTGAATCCCATAGAGAAA*	0,4 µM	48.8°C
Human PPM1B	Reverse	*GCACCCAAAGTATCGCCAGAA*	0,4 µM	57,8°C

Table S2. Antibodies

Antibody	Producer	Catalog Number	Antibody type	Dilution for WB
MYPT1	BD Transduction Laboratories	612165	Mouse,Monoclonal	1:500
Phospho-MYPT1 (Thr853)	Merck-Millipore	36-003	Rabbit,monoclonal	1:500
PRMT5	Merck-Millipore	07-405	Rabbit,monoclonal	1:1000
Phospho-PRMT5 (Thr80)	Abmart Inc	Sipos et al.	Rabbit,monoclonal	1:500
GAPDH	Santa Cruz Biotechnology	sc-47724	Mouse,Monoclonal	1:5000
PPM1B	Abnova	H00005495-B01P	Mouse,monoclonal	1:350
H2A	Merck-Millipore	07-746	Rabbit,monoclonal	1:1000
Dimethyl-H2A	Merck-Millipore	07-413	Rabbit,monoclonal	1:500
H4	Cell Signalling Technology (Massachusetts, USA)	L64C1	Mouse,monoclonal antibody	1:1000
Dimethyl-H4	Merck-Millipore (Darmstadt, Germany)	07-947	Rabbit,monoclonal antibody	1:500
pRB	Santa Cruz Biotechnology	sc-377539	Mouse,monoclonal antibody	1:1000
β-actin	Santa Cruz Biotechnology	sc-47778	HRP-conjugated	1:20000
anti-mouse	Cell Signaling Technology	#7076		1:5000
anti-rabbit	Sigma-Aldrich	A0545		1:5000

Table S3. Distribution of histological types

histological type	number of affected patients	percentage (%)
**adenocarcinoma**	21	55.26
**squamous carcinoma**	10	26.31
**microcellular carcinoma**	1	2.63
**macrocellular carcinoma**	1	2.63
**neuroendocrin carcinoma**	1	2.63
**necrotized granulomatosus infalammation. (non-TB)**	1	2.63
**colon cancer metastasis**	1	2.63
**sarcoidosis**	1	2.63
**gastric cancer metastasis**	1	2.63

Table S4. Distribution of pathological stages

pathological stage	number of patients	percentage (%)
T1bN0M0	2	6.25
T1cN0M0	5	15.62
T1cN1M0	3	9.73
T1cN0M0	1	3.13
T2aN0M0	9	28.12
T2aN2M0	2	6.25
T2bN0M0	1	3.13
T2bN1M0	3	9.73
T2bN2M0	2	6.25
T3N0M0	4	12.5

Supplementary figure legends

Figure S1. Overview of lung cancer tissue samples. (**A**) TNM classification of the samples is shown as a scatter plot, displaying the distribution of T (T1–T3) and N (N1–N3) stages across histological subtypes: squamous cell carcinoma (SCC) and adenocarcinoma (ADC). The M category was excluded from the figure, as all samples were classified as M_0. (**B**) Lung cancer tissue samples, categorized by their site of origin, are visualized on XY coordinates, where the X-axis represents the right and left lungs, and the Y-axis is divided into three sections corresponding to the upper, middle, and lower lobes. SCC is marked by write, ADC is marked by grey squares. (**C–D**) Kaplan–Meier survival curves illustrating overall survival (**C**) and disease-free survival (**D**) among lung cancer patients. Survival probability is plotted as a function of time (in months) from study entry. Patients were grouped by histological subtype: SCC (red) and ADC (black). Statistical comparison of survival curves was performed using the log-rank (Mantel–Cox) test. Significant differences. Where $p < 0.05$ (*) are indicated. The log-rank test statistic approximates the Chi-square value.

Figure S2. H4 symmetric dimethylation is downregulated due to the inavtivation of the PPM1B/MP/PRMT5 protooncogenic axis in lung adenocarcinoma (ADC) and squamous cell carcinoma (SCC). (A, B) Protein lysates derived from tumor and corresponding non-tumorous lung tissues of SCC and ADC patients were subjected to Western blot analysis using anti-H4 (A) and anti-symmetrically dimethylated H4 (DM-H4) (B) antibodies. Total H4 signals were normalized to GAPDH, while dimethyl-H4 signals were normalized to total protein levels. Quantitative results are presented as bar graphs showing mean ±SD (n = 3–6 per group). Group differences were analyzed using one-way or two-way ANOVA followed by Tukey's post hoc test,

and pairwise differences were examined with unpaired two-tailed t-tests. Statistical significance was defined as $p < 0.05$ (*).The original WB images are shown in Figure S11.

Supplementary Figures

Figure S1

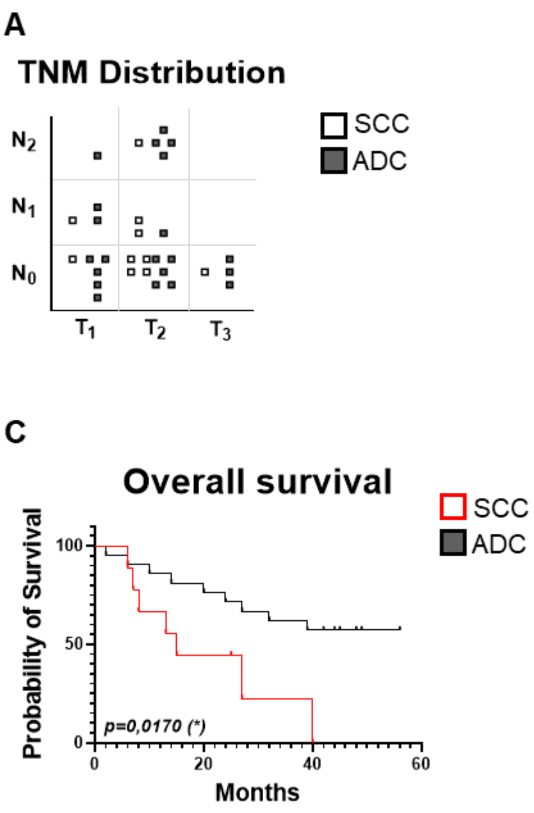

Figure S2

A

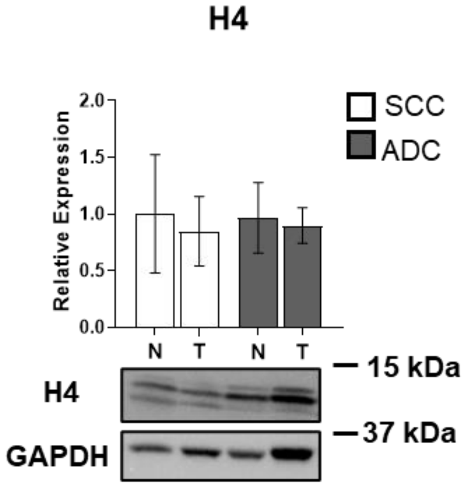

Supplementary blot
2023. 07. 26. H4

Supplementary blot
2023. 07. 26. GAPDH

B

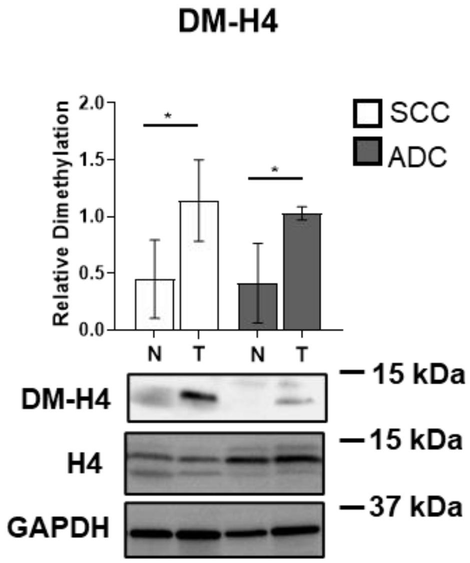

Figure S3.

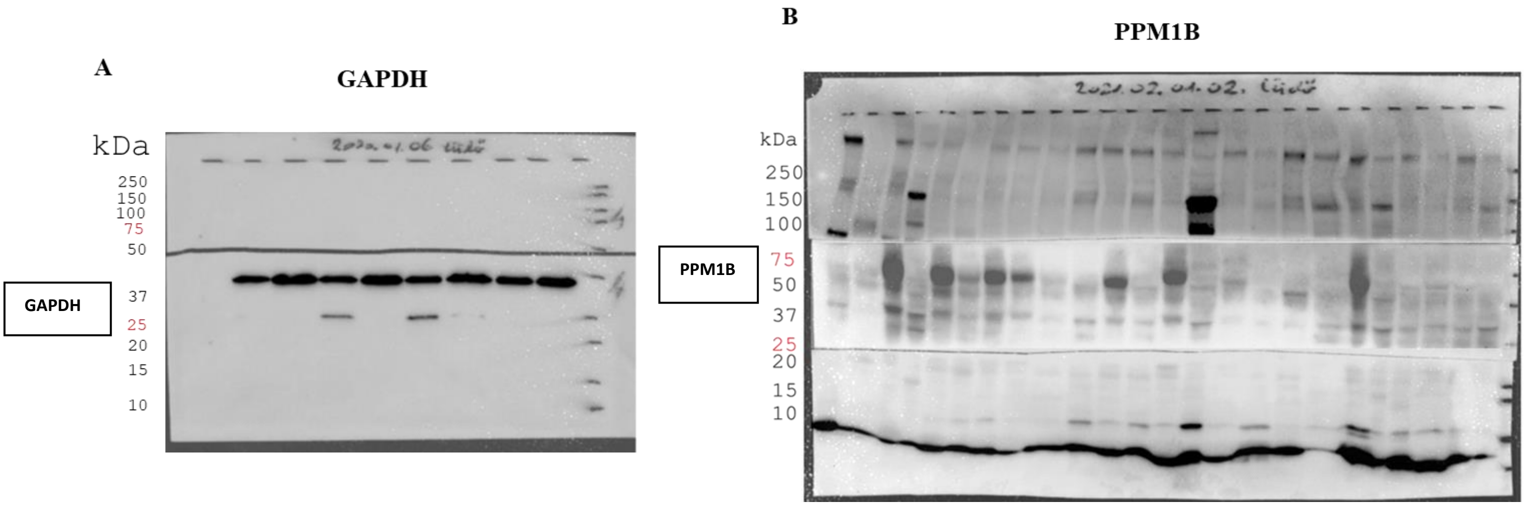

Figure S3. the original WB images of Figure 1A

Figure S4.

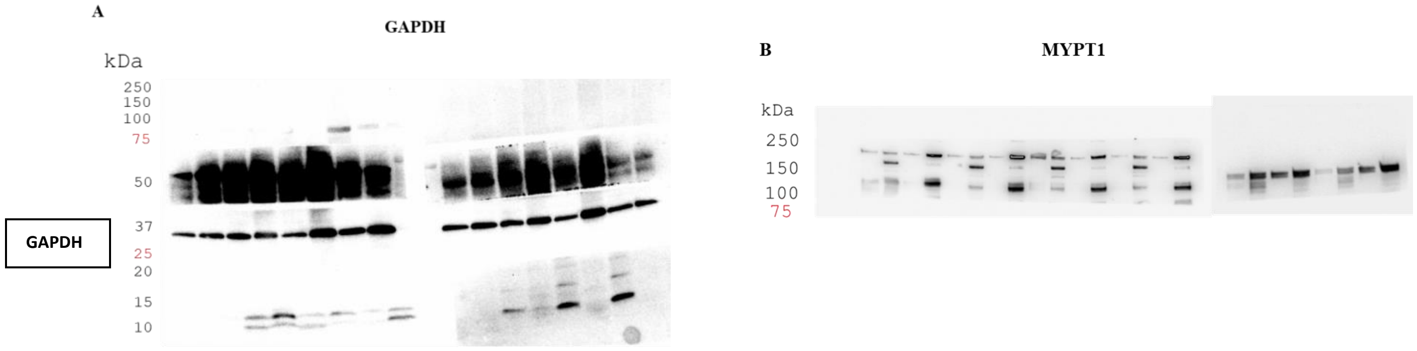

Figure S4. The original WB images of Figure 2A

Figure S5.

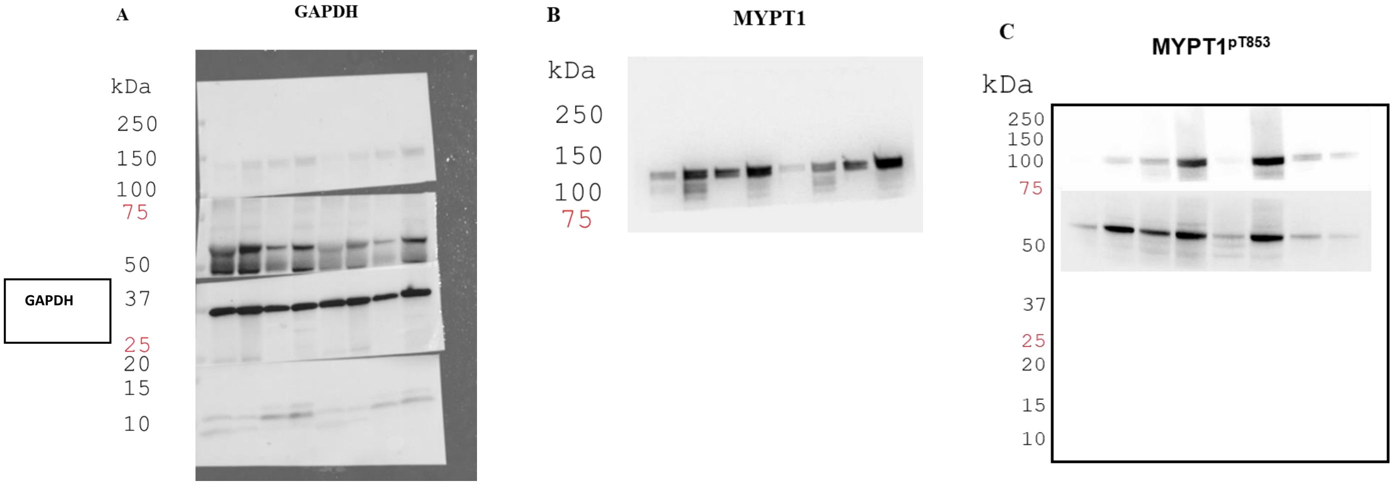

Figure S5. The original WB images of Figure 3A

Figure S6.

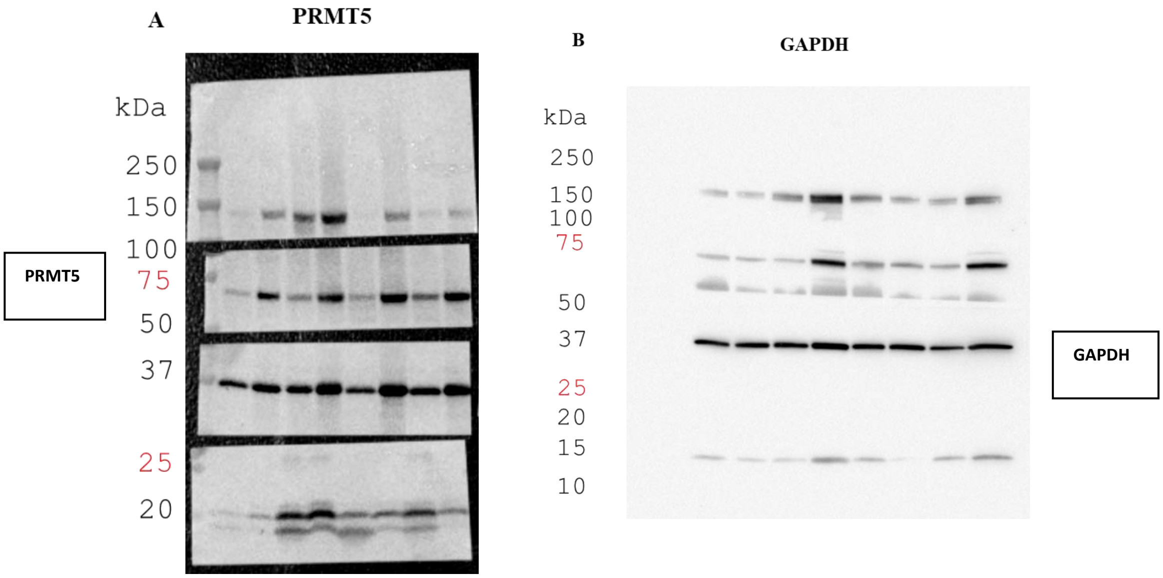

Figure S6. The original WB images of Figure 4A

Figure S7.

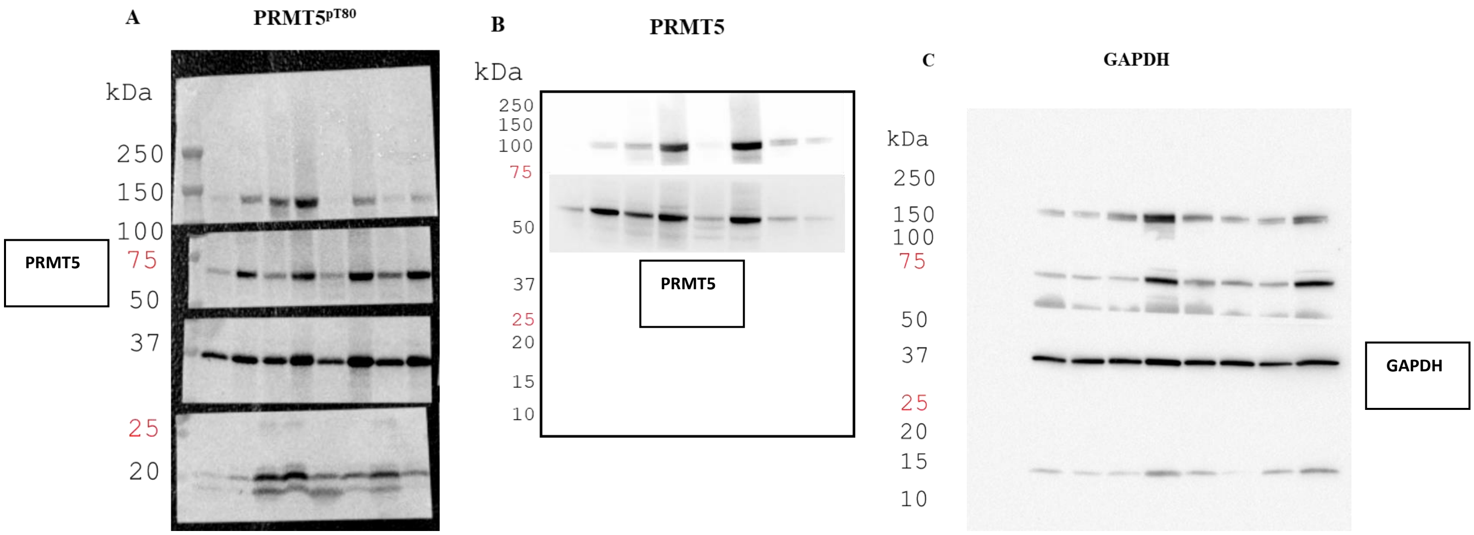

Figure S7. The original WB images of Figure 5A

Figure S8.

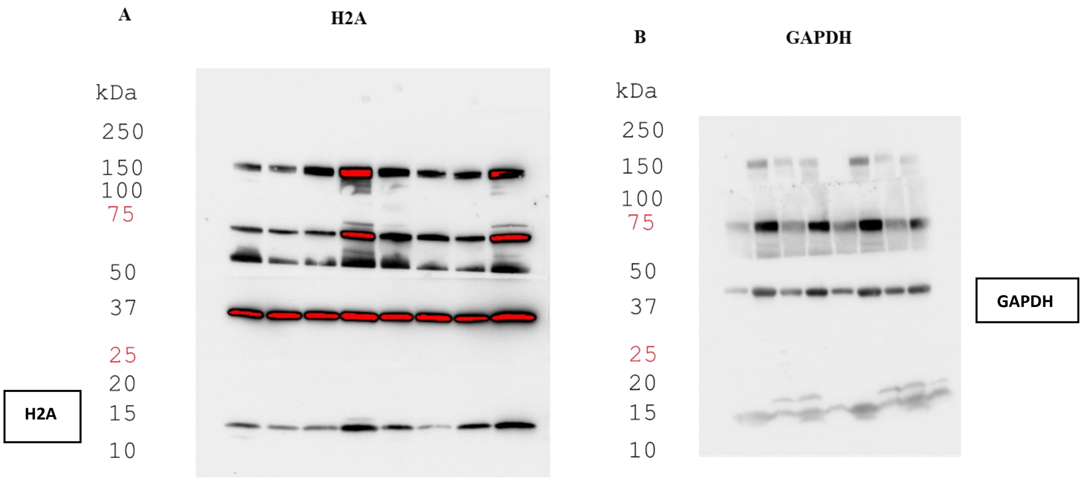

Figure S8. the original WB images of Figure 6A

Figure S9.

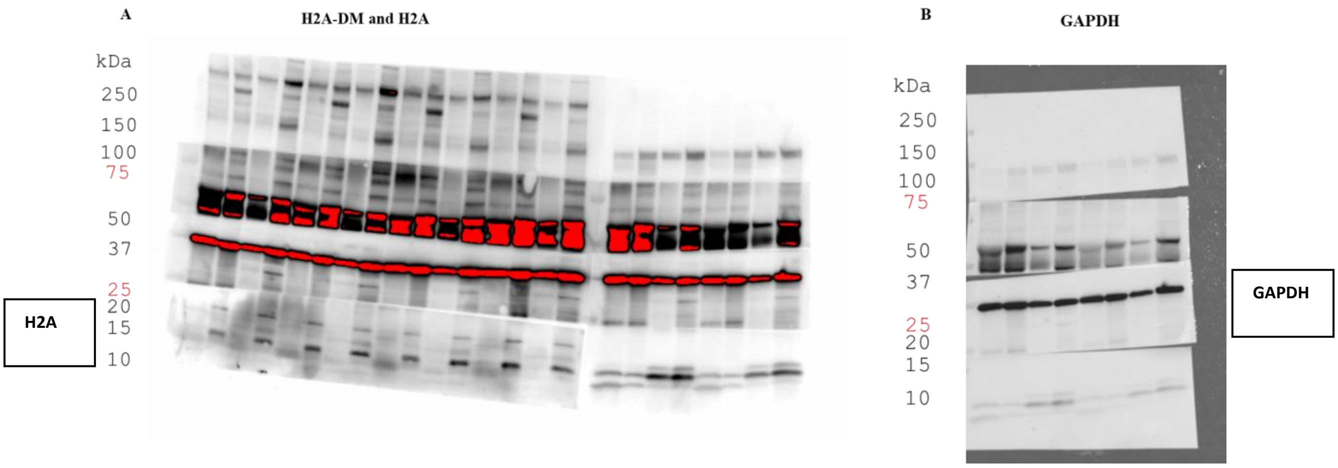

Figure S9. The original WB images of Figure 6C

Figure S10.

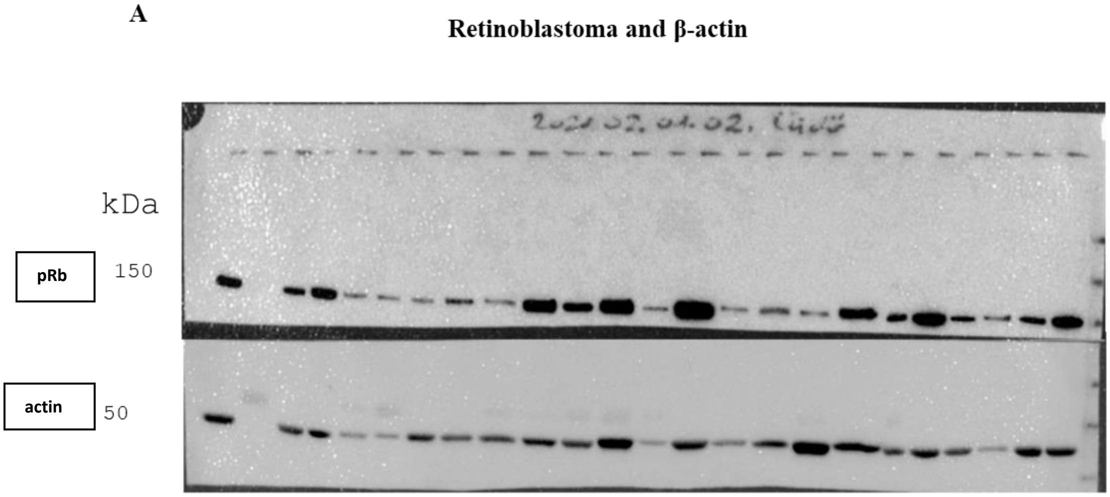

Figure S10. The original WB images of Figure 6E

Figure S11.

H4, DM-H4, GAPDH

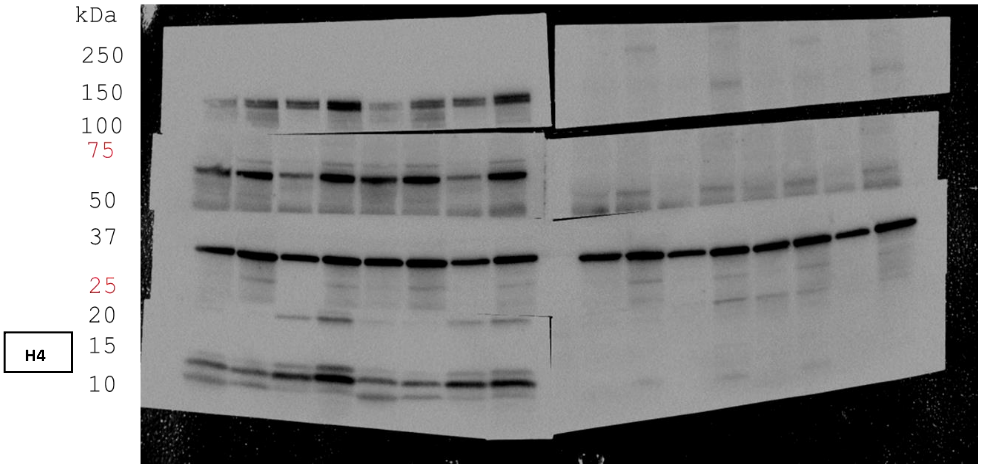

Figure S11. The original WB images of Figure S2A,B

Supplementary References

9. Travis, W.D.; Brambilla, E.; Nicholson, A.G.; Yatabe, Y.; Austin, J.H.M.; Beasley, M.B.; Chirieac, L.R.; Dacic, S.; Duhig, E.; Flieder, D.B.*, et al.* The 2015 world health organization classification of lung tumors: Impact of genetic, clinical and radiologic advances since the 2004 classification. *J Thorac Oncol* **2015**, *10*, 1243-1260.

37. Blumenthal, G.M.; Karuri, S.W.; Zhang, H.; Zhang, L.; Khozin, S.; Kazandjian, D.; Tang, S.; Sridhara, R.; Keegan, P.; Pazdur, R. Overall response rate, progression-free survival, and overall survival with targeted and standard therapies in advanced non-small-cell lung cancer: Us food and drug administration trial-level and patient-level analyses. *J Clin Oncol* **2015**, *33*, 1008-1014.

51. Asamura, H.; Chansky, K.; Crowley, J.; Goldstraw, P.; Rusch, V.W.; Vansteenkiste, J.F.; Watanabe, H.; Wu, Y.L.; Zielinski, M.; Ball, D.*, et al.* The international association for the study of lung cancer lung cancer staging project: Proposals for the revision of the n descriptors in the forthcoming 8th edition of the tnm classification for lung cancer. *J Thorac Oncol* **2015**, *10*, 1675-1684.52.

52. Yue, D.; Liu, W.; Chen, C.; Zhang, T.; Ma, Y.; Cui, L.; Gu, Y.; Bei, T.; Zhao, X.; Zhang, B.*, et al.* Circulating tumor DNA predicts neoadjuvant immunotherapy efficacy and recurrence-free survival in surgical non-small cell lung cancer patients. *Transl Lung Cancer Res* **2022**, *11*, 263-276.

53. Mazzone, P.J.; Lam, L. Evaluating the patient with a pulmonary nodule: A review. *JAMA* **2022**, *327*, 264-273.
